# How transdisciplinary research teams learn to do knowledge translation (KT), and how KT in turn impacts transdisciplinary research: a realist evaluation and longitudinal case study

**DOI:** 10.1186/s12961-023-00967-x

**Published:** 2023-03-21

**Authors:** Mandy M. Archibald, Michael T. Lawless, Maria Alejandra Pinero de Plaza, Alison L. Kitson

**Affiliations:** 1 National Health and Medical Research Council Transdisciplinary Centre of Research Excellence in Frailty Research to Achieve Healthy Ageing, Adelaide, SA Australia; 2 Caring Futures Institute, College of Nursing and Health Sciences, Flinders University, Bedford Park, SA 5042 Australia; 3 College of Nursing, Helen Glass Centre for Nursing, University of Manitoba, 99 Curry Place, Winnipeg, Canada

**Keywords:** Transdisciplinary, Knowledge translation, Translational medical research, Collaboration, Realist evaluation, Case study, Qualitative research, Mixed methods

## Abstract

**Background:**

Transdisciplinary research and knowledge translation are increasingly regarded as key concepts underpinning applied research across the health and social sciences, due to their presumed potential in addressing complex, “wicked” problems and improving the use of research in practice and policy, respectively. Despite sharing an impact mandate, the relationship between transdisciplinary research collaboration and knowledge translation remains unclear. In response, we examined the relationship between transdisciplinary collaboration and knowledge translation to generate these understandings with a view towards maximizing the impact of collaborative efforts.

**Methods:**

We undertook a realist evaluation and longitudinal case study of a 5-year National Health and Medical Research Council-funded Centre of Research Excellence in Transdisciplinary Frailty Research. Data were collected between February 2017 and March 2020 over three rounds of theory development, refinement and testing using interviews, observation, document review and visual elicitation as data sources. The Human Research Ethics Committee of the University of Adelaide approved this study.

**Results:**

Iterative analysis of narrative interviews and visual data led to the development of three overarching programme theories explicating the reciprocal relationship between KT understandings and transdisciplinary team process. These programme theories revolve around the concept of a network, which we define in alignment with extant theoretical literature on network mechanisms and complex networks as graphically representable networks of agents/people (nodes) joined by social relationships (links). Our findings demonstrate that under the right contextual conditions, transdisciplinary team members respond through an improved ability to (1) navigate the network, (2) negotiate the network and (3) mobilize the network.

**Conclusions:**

This research demonstrates the reciprocity and mutually supportive relationship between transdisciplinary research and knowledge translation. Our findings suggest that embedding a collaborative knowledge translation framework and providing resources such as facilitation and distributed leadership within a transdisciplinary team can improve collaboration and support transdisciplinary research objectives.

**Supplementary Information:**

The online version contains supplementary material available at 10.1186/s12961-023-00967-x.

## Background

The potential impacts of transdisciplinary research on the health sector and society at large are pronounced, and are of growing interest to policy-makers, funding institutes and researchers across disciplines [1–3]. The term “transdisciplinary” was introduced in the 1970s to describe a “*higher stage of succeeding interdisciplinary relationships which would not only cover interactions or reciprocities between specialized research projects but would place the relationships within a total system without any firm boundaries between disciplines*” [4] (p. 138). Since its introduction, interest in transdisciplinary research has grown remarkably: an electronic library search with the key term transdisciplinary yields 482 results for the year 2000, 2538 results for the year 2010 and 8168 results in 2020.

The growing emphasis on transdisciplinary research can be seen in the objectives and strategic priorities of leading national funding bodies such as the Australian National Health and Medical Research Council (NHMRC), the National Institutes of Health in the United States of America, and the Canadian Institutes of Health Research (CIHR) in Canada. Transdisciplinary research team grants and associated federal training initiatives are plentiful, as evidenced by the various initiatives of the National Institutes of Health (http://grants.nih.gov/grants/guide/rfa-files/RFA-CA-10-006.html), CIHR’s Strategic Training Initiative in Health Research emphasizing transdisciplinary research teams (e.g. Transdisciplinary Understanding and Training on Research—Primary Health Care) [5] and the NHMRC Medical Research Future Fund. Yet, the benefits of transdisciplinary research, including its ability to generate new knowledge and implement it to improve health, service delivery and complex social problems, are often implicit rather than directly attributed to the ways that the teams learn to work together. Understanding the relationship between transdisciplinary research and impact, and specifically to the concept of knowledge translation (KT) that is pervasive in the broader health system context, is therefore critical to better understand and inform how transdisciplinary teams work together to achieve research impact. The current inquiry seeks to investigate this relationship as it plays out in the context of a 5-year NHMRC-funded transdisciplinary Centre of Research Excellence (CRE).

In this study, the programme of interest involved a transdisciplinary network of investigators brought together to understand and mobilize knowledge around frailty and frailty screening in a transdisciplinary CRE. Like other transdisciplinary groups, the CRE existed within a health system emphasizing the translation of research evidence through KT—a concept commonly defined as the exchange, synthesis and ethically sound application of knowledge to improve health services, products and outcomes and strengthen the healthcare system [6]. Integrated KT (iKT), wherein knowledge users work with researchers to improve the relevance and uptake of research [7–9], is considered a particularly promising approach to bridging the gap between research and its use by way of stakeholder involvement to create the desired change in health systems. The emphasis on iKT reflects a shift towards engagement and the inclusion of perspectives that may traditionally fall beyond the scope of disciplinary knowledge, with potential to push understanding of partnership expectations and associated impacts or outcomes. Indeed, while the relationship between transdisciplinary research and KT is regarded with promise, the empirical and theoretical basis of such a relationship has not been fully explored. As such, we conducted a realist evaluation of a CRE—a naturalistic transdisciplinary research programme imbued with KT resources (e.g. facilitation, input from KT and implementation science leaders)—to inquire into the nature, and evolution, of such a relationship.

### Transdisciplinary research and “wicked” problems

Transdisciplinary research is collaborative research undertaken by investigators from different disciplines to generate novel approaches, theories, methodologies, products, programmes and/or policies [10, 11]. Rosenfield offered a foundational typological differentiation between multidisciplinary research, which involves independent research of various disciplines on a shared issue; interdisciplinary research, which involves the sharing of distinct disciplinary perspectives towards a shared problem; and transdisciplinary research, which involves the generation of a new model that transcends what was possible within any singular discipline [12]. This perspective of transdisciplinary is aligned with Piaget’s wider usage of the term as a *unity* of knowledge beyond disciplinary boundaries [4]. Correspondingly, transdisciplinary research is assumed to enable new perspectives, solutions and research impacts not otherwise possible.

As such, transdisciplinary research is regarded as integral to addressing “wicked” problems, such as chronic disease management, pandemic management and climate change. Such problems are complex, elusive to definition and contingent on the methods used to address them [13]. While interorganizational and multisectoral partnerships are necessary to address such problems, understanding how knowledge moves within and between teams to create system change is also critical [9, 14]. This requires an understanding of transdisciplinary research processes [15, 16] and attention to the complex relationships of transdisciplinary research with other concepts, such as KT, which share a number of foundational assumptions.

The expectation that research evidence will inform practice and policy has increasingly become the norm [7]. This impact sentiment drives both transdisciplinary research and KT science [2], which are trending towards a conceptual framework rooted in complexity science [9, 17–19]. Integral to each approach is an early alignment of priorities and a mobilization of networks, which contrasts with historically dominant conceptualizations of KT as a linear, rational and largely cognitive process [20, 21]. Yet, how academic communities understand KT can differ extensively, create so-called hidden barriers to successful KT due to epistemological differences [22], and consequently influence team function [23]. Without attention, there is a risk in assuming that the mere existence of partnership will improve mutual understanding towards shared objectives. Wicked problems require concerted attention to these epistemological underpinnings within a complexity-based approach.

Complexity-based approaches are supported by emerging frameworks and theories recognizing health system unpredictability—among other factors—within a whole systems view [9, 17]. For instance, Kitson and colleagues use complexity and network concepts to conceptualize the KT Complexity Network Model (KT-cnm), which is predicated on five interdependent subnetworks of key processes spanning problem identification, knowledge creation, knowledge synthesis, implementation and evaluation [9]. Activating the effective creation and movement of knowledge within this system is largely contingent on the agents promoting the change. The importance of actors within complex networks was also explored by Long and colleagues [24], who detail the network role concepts of central actor (e.g. key player with high degree of interaction within team) and broker (e.g. actor who plays a role in linking individuals within and to a team) in their study of leadership in translational networks, in reference to Gray’s [25] insights into the complexities of transdisciplinary leadership. While acknowledging the extensive literature on leadership in transdisciplinary team science, Stokols and colleagues identified interpersonal factors such as team diversity and analytical scope of inquiry that facilitate or inhibit transdisciplinary inquiry [26]. Such factors point to the range of “vantage points that must be bridged” (p. S99) through facilitation [26], which often require new methods or approaches to be developed to integrate such knowledge across disciplinary boundaries [2]. Whether KT can be a facilitative factor in transdisciplinary research remains uncertain.

This question became of central interest to our inquiry. At project conceptualization, the cocreating KT (co-KT) framework [8] underpinned the proposed approach. This co-KT approach is well aligned with iKT perspectives which position stakeholders in dialogue to advance mutual understanding from project outset, but reflects differences in historical development including the geographical locale of its development (e.g. iKT as a Canadian concept situated and supported through CIHR funding mandates; co-KT similarly emphasizing the democratic and iterative processes of production but devised in response to a “know-how” gap in doing KT within a population health framework in Australia) [7–9]. However, as the projects progressed, complexity science, including the KT-cnm, were increasingly considered in order to explicate the real-life messiness of how knowledge is constructed and moved through evolving and uncertain systems over time [17]. As our team adopted a way of working that involved KT theory, stakeholder engagement, and capacity-building of both junior KT leaders and KT capacity of other team members, as well as facilitation to attend to these aims, we became increasingly interested in understanding the relationship between transdisciplinary team research and KT, using an evolutionary (e.g. longitudinal) perspective, guided by realist evaluation.

### Realist evaluation

Realist evaluation seeks to explicate if, how, for whom and under what circumstances programmes work or do not work in different contexts [27–29]. It is well aligned with complexity-informed approaches, as contingencies are recognized as interacting, activating and capable of exerting myriad effects. Here, researchers seek to undercover how contextual (C) factors work with underlying mechanisms (M) to generate particular outcomes (O). Resulting C + M → O configurations represent explanatory pathways underpinned by implicit theories that can be made explicit through the realist evaluation process. We adopted this logic to understand the relationship of transdisciplinary research and KT. The programme of interest to this evaluation was a transdisciplinary CRE imbued with KT expertise and underpinned by an explicit KT theory, and is described in detail below.

## Methods

### Aim

#### Preliminary aim and initial programme theory

During project conceptualization we developed three questions, delineated in our protocol [1]: (1) Does transdisciplinary collaboration impact KT, and if so, by which mechanisms is this achieved? (2) What contextual factors determine whether the identified mechanisms produce their intended outcomes? (3) In what circumstances (e.g. combination(s) of context factors and mechanisms) are transdisciplinary teams most likely to be effective in terms of impacting KT? To generate an initial programme theory (IPT), we conducted a literature review and generated the working hypothesis that transdisciplinary research teams, combined with low-level facilitation, and implemented within a favourable team environment (C) will contribute to a shared perspective of KT as a complex, collaborative and iterative process (M), and be reflected in collaborative behaviours and attitudes in line with this perspective (O). We conceptualized low-level facilitation as activities led by KT investigators (MA, ML, AK) designed to feed back the team’s understanding and interpretation of KT within the context of their own research as well as the larger CRE network, including activities such as coproduction, stakeholder engagement and dissemination. Subsequent data collected focused on further developing, testing and refining the programme theory.

Our longitudinal approach resulted in a subtle shift in our aims—a shift that reflected the inherent complexity of working within and between the conceptual boundaries of KT and transdisciplinary research. Emerging conceptual contingencies challenged our own linearity of thinking (e.g. how transdisciplinary research impacts KT), encouraging a more cyclical and contingent conceptual model (e.g. KT and transdisciplinary research are mutually informative and conceptually related, and exert effects on each other). As such, following our IPT, we clarified our aims—maintaining a focus on the relationship between transdisciplinary research and KT—but with a renewed interest in exploring if and how transdisciplinary teams extend their thinking to embrace (or not) a working and integrated understanding of KT and how this understanding can influence transdisciplinary team process as a resourced aspect of team context. This reflected a theoretical shift from a co-KT orientation towards the complexity-based KT-cnm orientation.

### Design

This study was a realist evaluation with an embedded longitudinal case study of the relationship between transdisciplinary research and KT within the CRE over its funding lifespan (2015–2020). The programme that we were exploring was a transdisciplinary research team with a dedicated KT focus, enabling us to examine how transdisciplinary and KT processes influenced each other. As a result, we sought to gain insight into how transdisciplinary teams learned how to do KT and use it in an integrated way, and the consequential relationship/effect of this process on transdisciplinary research. Case study methodology seeks to generate deep and multifaceted understandings of an issue in a real-life context [30]—at times for the purpose of theory-building [31]—and as such is well aligned with the real-life, pragmatic orientation of realist evaluation [27–29].

### Context and setting

This study took place within a 5-year NHMRC CRE in Transdisciplinary Frailty Research to Achieve Healthy Ageing based in Adelaide, South Australia. The CRE comprised eight chief investigators (CIs), three associate investigators (AIs), four international AIs, 12 research fellows and seven graduate students, bringing together expertise in medicine (geriatrics, gerontology, general practice), KT, nursing, demography, orthopaedic surgery, pharmacy and health economics. It included interstate and international collaborators and advisement from a panel inclusive of governmental, community and lay representatives. The CRE adopted a multilevel approach to frailty research. The funded grant focused on (1) describing and mapping the frailty burden, (2) developing a new economic model for frailty, (3) identifying risk earlier in the community and (4) developing a cost-effective model for frailty intervention [32]. While the CRE commenced during grant writing (before 2015), the team was further developed with the recruitment of a postdoctoral KT fellow in August 2016 (MA) to work with KT leader and CI (AK). At this time, the co-KT framework [8] underpinning the CRE was operationalized through a stakeholder-driven approach involving (1) understanding perspectives of key stakeholder groups regarding frailty and frailty screening [33–35], (2) incorporating these perspectives in other domains of inquiry and activities initiated by other investigators [36] and (3) developing creative and collaborative KT resources to support public and healthcare provider understandings [37, 38] using a range of engagement methods.

#### Context resources

Within realist evaluation, context resources (i.e. resources available through a programme) enable responses from actors and influence programme outcomes. Notable context resources of the CRE included the use of an explicit KT framework underpinning the CRE’s four research directions; the leadership of a KT expert (AK) as CI and an emerging KT scholar with expertise in arts-based, creative and collaborative KT (MA); and the availability of KT investigators to consult and collaborate on team research activities and facilitate KT capacity-building activities. A series of activities were undertaken from August to September 2016 including meetings with CIs and other CRE investigators to establish professional relationships and personal connection, discuss potential areas of overlap and opportunities to generate a research vision, and introduce and emphasize the relevance of KT to the overall project initiatives. An overview of the activities comprising facilitation is provided in Additional file 1. The structure, approach and composition of the CRE resulted in an embedded approach to KT wherein the understandings and behaviours of investigators related to KT could be studied and facilitated internally, in concurrence with and as a by-product of the transdisciplinary team composition.

### Data collection

We collected data from the following five sources: (1) document review, (2) narrative data from semi-structured interviews, (3) numerical data from publication and grant outputs, (4) visual data from participant-elicited drawings and (5) observation, reflective and analytical memos. Data were collected between February 2017 and March 2020, over three rounds of theory development, refinement and testing [1]. We describe each data collection period below, in addition to the initial document review that contributed to our IPT and construction of the semi-structured interview guide. The initial document review and IPT development methods are briefly described in our study protocol [1] but are expanded upon here, structured by its use in each theory development stage (IPT development, elicitation, verification, refinement).

#### Devising the IPT using document review

Prior to the baseline data collection (round 1), we reviewed the NHMRC CRE grant application, the CRE website and meeting agendas, and peer-reviewed and grey literature relevant to KT, research collaboration, transdisciplinary teams, organizational management and high-performing teams. MA scoped databases and grey literature (Open Grey and Google Scholar) to identify potentially relevant articles and devise an IPT. This subsequently informed the search strategy, which ML and MA developed in conjunction with a university research librarian. We aimed to identify English full-text empirical, critical or interpretive articles of any design, published between January 2000 and July 2017, that were available online and that reported on team-based science and/or research collaboration between academic disciplines; interventions or strategies to support KT-related understandings and behaviours and/or transdisciplinary research collaboration; and processes of connecting knowledge generation to knowledge use in any discipline(s) or setting. We adapted the initial search strategy to include three databases (PubMed, CINAHL, Scopus—Additional file 2) and the grey literature. MA and ML performed lateral searching, including forward and backward citation tracking and reference list searching. The search was updated in May 2018 (round 2) and December 2019 (round 3). The search strategy was iteratively modified over each data cycle and results were uploaded into EndNote X7 (Clarivate Analytics, PA, USA).

Through this process, we constructed an IPT of the relationship between transdisciplinary research and KT [1]. Here we broadly situated the concepts of facilitating, interpreting, aligning and engaging as possible outcomes influenced by higher-level CRE contextual factors across political, social, environmental and organizational domains. We postulated that internal (e.g. leadership) and external (e.g. resources, outputs) measures of functioning would be supported by these processes and contexts, and would be influenced by the various disciplinary interactions of the CRE.

#### Data collection period 1: development and elicitation

- Between February and March 2017, we explored relevant theoretical constructs (context-mechanism-outcome [CMOs]) through interviews and visual elicitation.
- Using an initial interview guide based on the IPT and informed by consultation with team members [1], MA conducted semi-structured face-to-face or videoconference interviews [39] with CRE team members (sample described below). The interview guide inquired about the participants’ background and disciplinary upbringing; perceived role within the CRE; how their background influenced the nature of engagement in the CRE; the collaborative nature of projects each participant was involved in (including their genesis); experiences of existing CRE structures (e.g. management, location, resources); perceptions of communication within the CRE; motivating and demotivating factors within the CRE; perceived objectives of programme stream and overarching CRE and KT goals; perceptions of what is required to achieve these stated goals and objectives; perceptions of KT including what has worked and what has not worked; meanings of evidence; perceptions of KT and their origins; the relationship of transdisciplinary research to KT; and other factors identified as pertinent to transdisciplinary team function and KT in the IPT.
- MA conducted visual elicitation alongside the interviews to garner insight into participants’ representations and associated sense-making processes [40–42]. Visual elicitation involves the use of visual media or representational forms, such as drawing or photography, to stimulate participant discussion and to provide different or deeper insights into the research phenomena [42]. In each interview, participants were provided with two blank A4 pieces of white paper and a pencil. They were asked to illustrate their understanding of KT and then how transdisciplinary research relates to KT to generate a baseline understanding of team members’ narratives and perceptions (e.g. mental models). The timing of this activity occurred at the approximate midpoint of data collection period 1, enabling participants to engage in traditional interviewing, which may be more familiar to their previous experiences.
- MA and ML observed scheduled CRE events and meetings whenever possible, including monthly management meetings and mentoring events. As participant observers [43], we recorded our observations of activities and interactions as field notes. This approach was used throughout the study duration to provide additional context related to the emerging programme theory, and to inform reflexive discussions between members of the KT team.

#### Data collection period 2: verification

- Between March and June 2018—the midpoint of CRE funding—we conducted semi-structured interviews with participants and verbally presented working programme theories to participants in order to amend or refute our working hypotheses.
- Participants were able to revisit their drawings from the first data collection period and modify a superimposed copy of the drawing, if desired. Through these means, participants were able to clarify, amend or refute their initial conceptualizations and working hypotheses.

#### Data collection period 3: refinement

- Between February and March 2020—the final year of CRE funding—we presented our working programme theories to participants in order to amend or refute working hypotheses. We presented the theories using visual aids (e.g. illustrated depictions of working hypotheses) and interactive tasks (e.g. asking participants to rank outcomes of transdisciplinary collaboration in order of perceived importance).
- We conducted case-by-case productivity mapping (e.g. number of publications and grants per investigator per year) and citation tracking/counting to enable pre- and post-CRE comparisons of productivity and research impact (i.e. engagement with healthcare consumers, policy) to further augment our understanding of the relationship between transdisciplinary research and KT. A summary of key milestones of the CRE is presented in Table 1.

**Table 1:** Milestones in the CRE life cycle (2015–2020)

Milestone	Date
Official launch of the CRE Frailty and Healthy Ageing	September 2016
KT emphasis on co-KT Several postdoctoral fellows and graduate students join the CRE	September 2016–May 2017
Mentoring committee established	February 2017
Realist evaluation data collection time point 1	February–April 2017
CRE planning workshop	April 2017
CRE research showcase	September 2017
Realist evaluation data collection time point 2	March–June 2018
CRE research showcase	May 2018
KT planning workshop	July 2018
Further consideration of KT-crn Consumer focus group workshop	Aug 2018–September 2018
Video resource codesign workshops	December 2018–March 2019
PRAXIS art exhibition	April 2019
Realist evaluation data collection time point 3	February–March 2020
Presentation of preliminary findings of realist evaluation at CRE management meeting	September 2020
Final CRE research report	September 2020

### Sample

We sampled across CRE subgroups, inviting all Australian-based CIs and AIs and international AIs to participate, and purposively sampled postdoctoral research fellows and graduate students based on their extent of activity and role in the CRE. We sought to include the same investigators throughout each data collection phase. Initially, we sought out crucial cases (i.e. cases critical to refining and testing the IPT). As we progressed, we paid increasing attention to typical cases (i.e. cases most likely to represent the IPT; investigators in close contact with leading CRE members and activities) and deviant cases (i.e. cases most likely to contradict the IPT, such as off-site participants less exposed to collaborative efforts) [44]. Through frequent reflexive discussions within the KT team at project conceptualization and initiation, we chose not to include the KT investigative members (AK, MA, ML, AP) as study participants, recognizing the significant role that we were playing in facilitating transdisciplinary and KT cohesion—namely, AK as the lead KT investigator and developer of the co-KT and KT-cnm approaches; MA as lead operational investigator of the stakeholder, creative KT-driven approach, and ML and AP as KT investigators who, with MA, led facilitation of the KT team. We recognized these key facilitative roles as imperative to shaping how the CRE responded to KT concepts, influencing transdisciplinary research and influencing the study outcomes.

### Data analysis

Iterative data analysis occurred after each data collection cycle to advance from theory elicitation through to verification and refinement. The main analysis structure involved mapping the data by activities and programme modalities of the CRE and context, agents/actors (e.g. individuals, groups or institutions influencing and/or affected by programme outcomes), mechanisms (i.e. how actors respond to programme resources to produce various outcomes) and outcomes (e.g. KT behaviours and outputs) in relation to the IPT. Like Dalkin et al. [44], we conceptualized mechanisms as comprising the *resources* introduced into the context and the *reasoning* of participants which lead to outcomes. We developed a standardized form to identify the relationships between these elements, mapping possible CMOs to our working hypotheses (Table 2) [i.e. *transdisciplinary research teams, combined with low-level facilitation from KT researchers (MA and AK) and implemented within a favourable team environment (C), will contribute to a shared perspective of KT as a collaborative, complex and iterative process (M), and be reflected in behaviours (e.g. communication and collaboration methods) and successful implementation of study findings in line with this perspective (O)*].The mapped CMO configurations were used as a basis for iterative CMO development and revision.

**Table 2:** CMO mapping template

Context Elements of both the physical and social environment that enable or impede the expected outcomes	Actor Individuals, groups or organizations involved in the implementation and outcomes of an intervention	Mechanism An explanation or justification of why a resource was used by an actor to achieve an expected outcome (or not)		Outcomes^a^ *Immediate* Immediate effects of CRE programme activities (e.g. changes in knowledge, attitudes, skills and awareness preceding changes in behaviour) *Intermediate* Intermediate outcomes of the CRE identified through indirect effects of CRE activities (e.g. changes in behaviours or practices following immediate changes in knowledge and skills) *Long term* Changes in the medium and long term, reflecting further indirect effects of CRE activities and less accountability of the programme elements (e.g. impacts on community and health system)
		Resource Resources and programme activities introduced	Response Participants’ reasoning in response to resources	

^a^Outcomes specified for analytical clarity also used to guide IPT

We used inductive and deductive reasoning to anchor the analysis to the building, refinement and testing of CMOs. MA and ML repeatedly read the interview transcripts and field notes before coding using a standardized framework. Data were analysed within and across sources and cases. As a deviation from our protocol, we focused observational data on narrative recordings and reflection rather than Spradley’s [45] dimensions, which proved unfeasible. Data were extracted and coded in a Microsoft Excel workbook. Concepts were charted across sources using concept mapping (4) and a three-step process to derive explanatory statements: (1) extracting units of data (e.g. interview excerpts) relevant to the IPT and constructing CMOs; (2) sorting the CMOs by concept to develop consolidated statements (i.e. groups of CMO configurations); and (3) collectively comparing and cross-verifying the consolidated statements to derive higher-order explanatory statements. Through frequent analytical meetings with the KT team, we frequently re-examined our positions to the research data and how our specific standpoints and attributes may influence the study findings. This process enabled us to explore points of complementarity, reciprocal resonance and/or refutation (e.g. through the purposive inclusion of “deviant” cases in the analysis) to refine the programme theory. Findings were verified with the larger CRE team members and stakeholders in August–September 2020 at CRE management and advisory group meetings, wherein methods and findings were presented and discussion was facilitated to consider alternative interpretations and processes at play.

Due to the nature of the visual data obtained, we considered the visual data in relation to the narrative data, deviating from the visual content analysis framework explicated in our protocol [1]. We viewed the visually elicited data as supplementary to the narrative data, as it was often used as a prompt for deeper insights and discussion; participants infrequently completed the drawing as a stand-alone work but rather supplemented it with their explanations. Aligned with our protocol, we considered most carefully the constituent elements (i.e. content), configuration (i.e. positioning of elements) and size (e.g. suggesting importance) of drawings; this visual data provided further insight into narratives and representations of complex concepts that might be inaccessible using exclusively verbal means.

In addition to the narrative and visual data, we considered productivity data of investigators’ publications, presentations and grant success. We regarded this as a component of understanding the effectiveness of the transdisciplinary team at promoting inter-team collaboration. While a coarse representation, these data also helped generate a view of the productivity and impact trends occurring—and for whom—within the CRE.

### Ethics

The University of Adelaide Human Research Ethics Committee gave ethical approval for this study (H-2016-284). Investigators were assured that their responses would be confidential and would not influence opportunities for collaboration within the CRE.

## Results

The findings are presented in two sections. First, the programme theories are introduced as three overarching statements, explicated by CMO descriptions. Second, each CMO is discussed with attention to corresponding sub-theories, which provide more granular detail explicating each programme theory. Supportive data for each CMO are integrated throughout. The Preferred Reporting Items for Systematic Reviews and Meta-Analyses (PRISMA) diagram, which shows the flow of article selection for the document review, is located in Additional file 3. Reporting of the realist evaluation is aligned with RAMESES (Realist and Meta-Narrative Evidence Syntheses: Evolving Standards) II, Reporting Standards for Realist Evaluation (Additional file 4).

### Programme theories

Narrative interviews and visual data led to the development of three overarching programme theories (CMOs 1–3) about the reciprocal relationship between KT understandings and transdisciplinary team processes. These programme theories revolve around the concept of a network, which we define with respect to extant theoretical literature on network mechanisms and complex networks, that is, a graphically representable network of agents/people (nodes) joined by social relationships (links). In the KT literature, networks comprising interconnected agents (e.g. organizations, people) are conceptualized as providing the social context in which translation and implementation efforts take place [46].

1. Navigating the network: Governance structures and processes—including facilitation—that enable team members to identify and connect existing resources in the network and to develop relationships with each other and with stakeholders (*context*) will increase team members’ perceived capacity to navigate the features, interrelationships and boundaries of the scientific or societal problem space to be addressed. This will also increase the CRE teams’ ability to contribute to positive perceptions of the CRE, foster trust and nurture a willingness to move towards unified goals and action plans (*mechanism*). This will result in a deeper understanding and awareness of the research problem and the local context of the research, strategies to synthesize and apply existing knowledge, and positive perceptions of collaboration and teamwork, influenced by a sense of belonging and shared purpose (*outcomes*).
2. Negotiating the network*.* Formal and informal collaborative arrangements and distributed/flexible leadership that give CRE members the opportunity to reshape relationships, goals, roles, communication and engagement approaches, and facilitate opportunities for feedback and reflection (*context*), will improve CRE team members’ ability to negotiate and leverage available network resources and support(s) and extend this to engagement with external organizations (*mechanism*). This will lead to enhanced team leadership ability, collaborative decision-making, tailored knowledge exchange opportunities and positive attitudes towards learning, reflection and adapting problem-solving and engagement approaches (*outcomes*).
3. Mobilizing the network. Access to systems, resources (e.g. funding, personnel) and strategies for constructive stakeholder interactions and facilitated reflective activities (*context*) will facilitate a shared KT agenda and shared understandings of how these goals can be achieved by mobilizing people and resources in the network (*mechanism*). This will result in team members developing an understanding of key KT concepts and processes, knowing how to share research findings with various stakeholders, having the capacity to interpret and use knowledge, and having increased confidence in their abilities related to team collaboration and undertaking various KT activities (e.g. leadership in KT projects when no previous KT expertise existed).

**Fig. 1:**
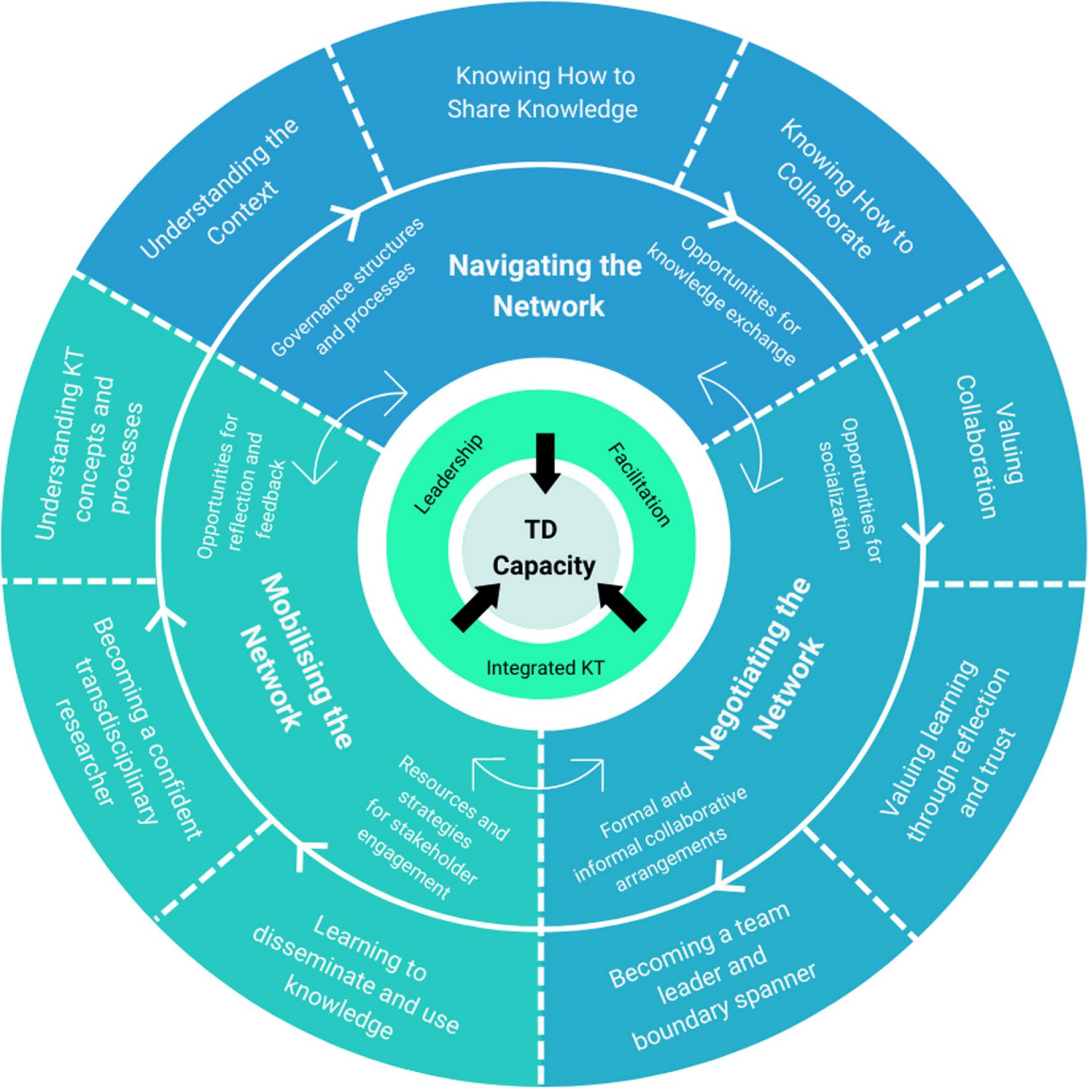
Visualization of the three overarching programme theories (CMOs 1–3). The outer circle represents the programme outcomes (O). The middle circle represents the context factors (C) and network mechanisms (M). The inner circle depicts the critical components or “active ingredients” of the programme that work together to influence individual and team-level transdisciplinary research capacity. TD = transdisciplinary; KT = knowledge translation. The bidirectional arrows indicate the interrelatedness contingent influences of the contextual factors and network mechanisms. The centre-pointing arrows indicate the direction of influence of these factors on the primary outcome of TD capacity

To aid understanding, we provide a visual representation of key contextual, mechanistic and outcome components in Fig. 1.

## Navigating the network

**Fig. 2:**
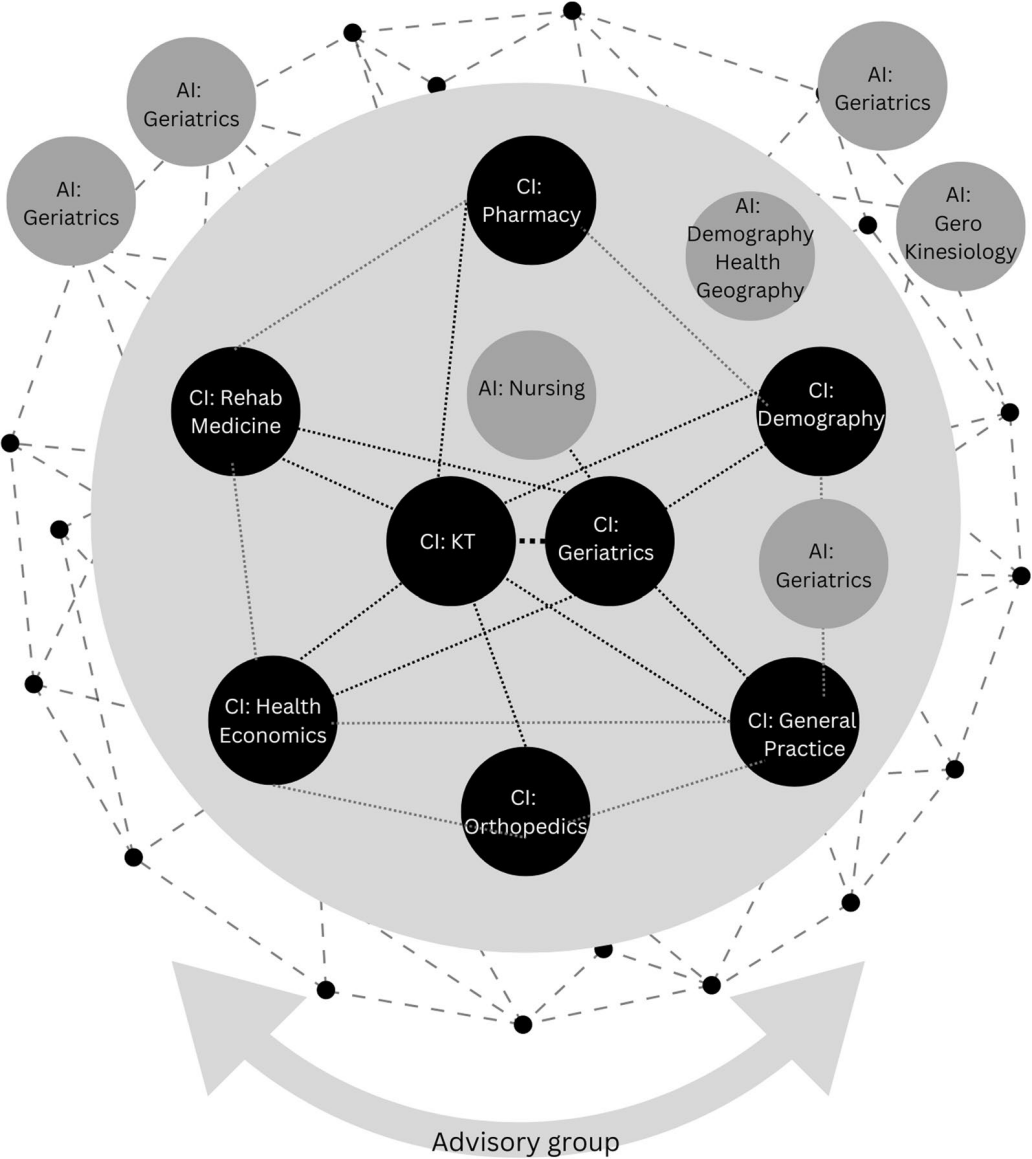
Internal CRE structure at conception. The outside solid circle represents the internal structure of the CRE, including Australia-based CIs and AIs and their respective disciplines. The colour of each circle reflects the designated role of the investigative leadership, with darker circles representing a higher level of assigned leadership. The dotted lines between members reflect the anticipated relationships between nodes. The outer network image reflects the orientation of the CRE towards societal impact. The circles outside within the network reflect international AIs, and the underpinning arrow reflects input from the advisory group

**Fig. 3:**
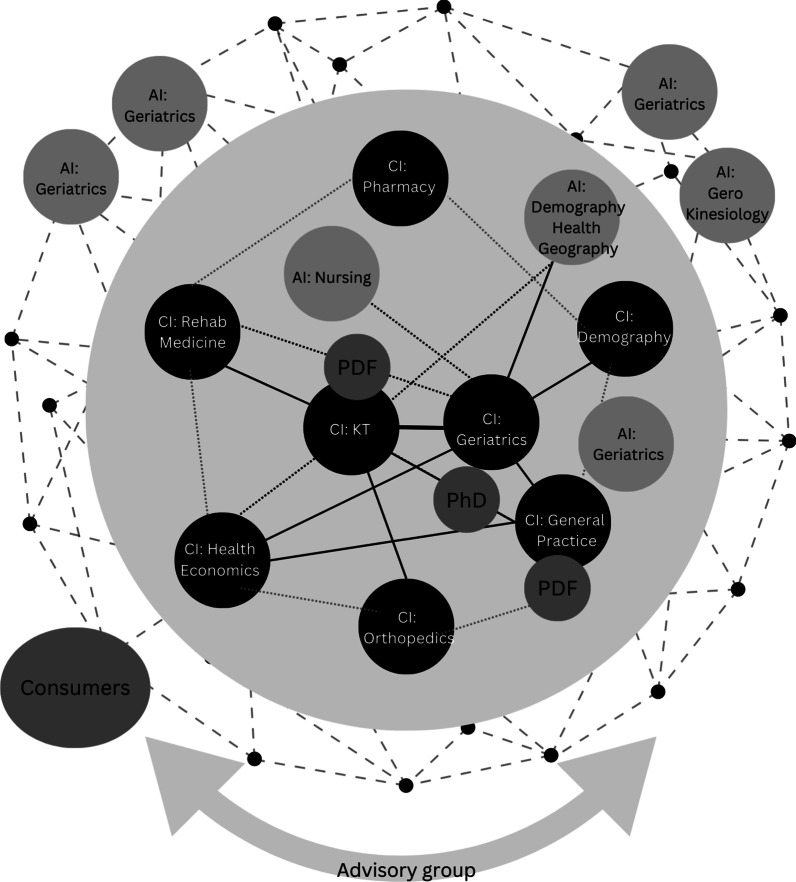
Internal CRE structure in year 2. The outside solid circle represents the internal structure of the CRE, including Australia-based CIs and AIs and their respective disciplines. The colour of each circle reflects the designated role of the investigative leadership, with darker circles representing a higher level of assigned leadership. The strength of relationships between investigative teams is indicated by darker line weight. At this stage, an emphasis on consumer perspectives became integral to the KT objectives. The outer network image expanded in size to reflect the broader social networking and impacts of the CRE over time

**Fig. 4:**
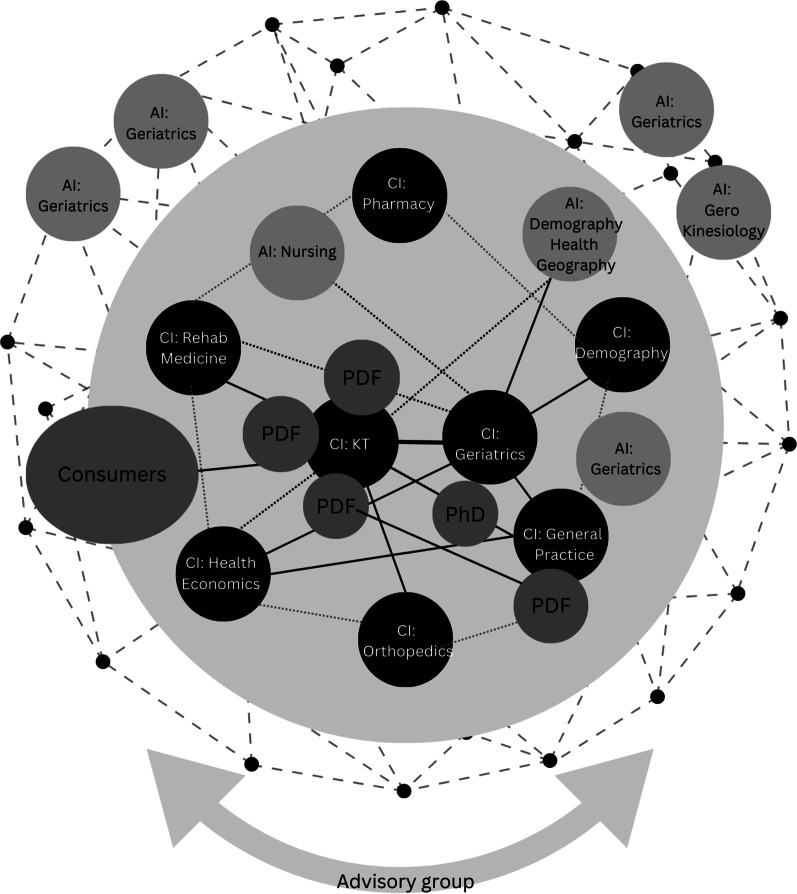
Internal CRE structure in year 3. The outside solid circle represents the internal structure of the CRE, including Australia-based CIs and AIs and their respective disciplines. The colour of each circle reflects the designated role of the investigative leadership, with darker circles representing a higher level of assigned leadership. The strength of relationships between investigative teams is indicated by darker line weight. At this stage, consumer involvement in the KT stream increased, enabling codesign approaches to be integrated and valued within the CRE. Consumer insights permeated other activities of the CRE and informed research directions. The influence of PDFs (postdoctoral fellows)increased and the relationships between them strengthened. This propelled progress towards shared objectives and strengthening relationships within the network. Additional CRE structures in years 4 and 5 reflected similar composition, with the removal of one PDF from the KT team

Team structure and associated collaborative processes influenced how team members identified and connected with existing resources, such as people, theories, methods, data, technology and equipment. Governance, team process, time—specifically the duration and frequency of exposure to new ideas—and opportunities for diverse engagement and knowledge exchange were key aspects of context activating investigators’ openness to engaging collaboratively. This also influenced the extent of boundary-crossing occurring within the team. Participants frequently mentioned navigating between agents and contextual resources in order to explore opportunities for knowledge exchange and collaboration. The following sections describe the interactions between these various contexts, mechanisms and outcomes that underpin the programme theory. Figure 2 provides a general visual overview of the internal CRE structure at the time of its conceptualization; Figs. 3 and 4 reflect how this structure changed over time.

### Understanding the context

Structures and resources for team members to engage with stakeholders and cocreate knowledge with users was an important component of context. This context enabled team members to recognize opportunities, constraints and barriers in the policy or practice settings (*mechanism*) and to work together to address them. As a result, team members were able to develop an understanding of “what goes on” and “how things work” in specific organizational settings, daily practice or local healthcare systems (*outcome*). These gains included knowledge about sociopolitical factors that could affect the application of new knowledge; the local, historical and/or political conditions and features of an organization or system; settings and organizations that are interested and open to research participation and use; basic knowledge about communication, public relations and marketing; gauging the applicability of evidence to other contexts; and the sustainability of research-based knowledge in different contexts. As one CI explained, “whatever we do policy-wise has to be acceptable, we have to be able to make sure it fits with the new paradigm of well-being. That’s the bit I think is important. It has to fit the paradigm” (Participant 1.7).

A foundational component of context was the leadership approach adopted at different levels of governance within the CRE. Through reflexive writing and discussion, we came to recognize that a highly distributed leadership style within the KT stream enabled a context favourable for autonomous practice and leadership development, wherein strategies developed and implemented were well aligned with investigators strengths. One CI commented, “If I want the final end to be something new, I have to let everyone go at their own pace and have trust that there will be something new at the end” (1.6). Other research streams that demonstrated distributed leadership also achieved notable boundary-spanning knowledge development (e.g. general practice stream). This strengthened the relationships between other actors in CRE, such as the KT stream (Figs. 2, 3, 4). Here, collaboration was a deliberate and active process: “In terms of looking for opportunities to collaborate, I think a good example of processes is [the general practice] project, because [it has] drawn in everyone from across the CRE” (CI, 1.9).

### Knowing how to share knowledge

Establishing opportunities for dialogue and knowledge exchange among team members and stakeholders and facilitating team-based reflective learning on knowledge-sharing approaches (*context*) enables team members to comprehend the views and needs of team members and stakeholders, determine the direction (or “pitch”) of the research and tailor the research accordingly (*mechanism*) (i.e. “making sure that the message actually resonates with people that you want to be the target audience”, graduate student, 1.3). As a result, team members, particularly those with high levels of exposure to facilitation and investment in the shared direction of the research team, develop the ability to share information with diverse stakeholders, including being able to conduct research of relevance to intended users so that it is more likely to inform policy and practice, cocreate knowledge with stakeholders, interpret data and make data-informed decisions to promote the use of research findings, and involve stakeholders in research processes (*outcome*).

Facilitation activities of the KT team, including the interviews conducted with participating investigators, provided opportunity beyond the assessment of investigators’ knowledge and attitudes towards KT. Rather, these encounters were often the first circumstance where investigators considered their views on KT, including what knowledge is and how it should be shared (e.g. “I haven’t really thought about [KT]”, 1.5, graduate student). By KT facilitators orientating the first phase of data collection towards the purpose of the CRE, its vision and what success means to each investigator, team members were provided with a framework to consider research impact. This was particularly notable for investigators with high visibility and investment within the CRE team, namely, CIs and their research trainees, when those trainees (e.g. postdoctoral fellows) were granted autonomy in research practice. The impact of facilitation activities was most notable for those clinically practising investigators or those working in clinical practice research domains—at CI, AI and trainee levels. As a CI expressed, “I might see someone who’s on our advisory group on an operational matter and go, ‘What do you think about this?’ and then we have a gossip session, but that’s actually KT. It’s codesign, it’s the next idea” (1.6). These individuals generally presented more openness to facilitation activities; knowledge exchange occurred more fluidly across respective disciplinary boundaries.

For interstate and international collaborators, particularly those with weaker ties with local points of contact (e.g. minimal record of copublication) and those in clinical roles, facilitation activities of the KT team were infrequent and typically limited to videoconference interviews or email exchanges. As a result, there were fewer opportunities for interstate and international researchers to discuss their activities, progress and perspectives on KT, leading to reduced investment in the shared objectives of the team. One interstate CI commented, “Probably the biggest difference is that I feel a greater sense of geographical separation for the current CRE, because there are other investigators who are not based in Adelaide, but I think they’re the minority…I’m used to, in a CRE setting, have more regular contact that’s somewhat more easily facilitated than in the current CRE.”

### Learning how to collaborate

Providing time, resources, structure and a supportive environment for team members to build connections and reflect on these connections (*context*) will enable team members to perceive which collaborations are going to be productive and effective (or not); and allow gaps, interdisciplinary or interprofessional tensions, and trust-based opportunities to surface in a planned way (*mechanism*). This in turn can lead to the ability of team members to successfully engage others (e.g. academics, stakeholders) in collaborative efforts.

A key mechanism activated through the resourced CRE context was trust. While trusting relationships were also observed as an outcome of effective collaboration, trust was something gained (or lost) through various means. For instance, frequent and persistent support for KT learning and concurrent openness on behalf of each party encouraged trust, enabling new research concepts, directions and collaborations to take hold. As one CI explained, “It’s a matter of trusting how someone else works and going, ‘I just gotta go with this a bit’” (12). For investigators with previous relationships, trust was something established through previous reputations or collaborative work (e.g. competence trust). Trust between new collaborators most often began as transactional, and over time through met-or-exceeded expectations, grew to trust of team members’ skills and abilities. As one student reflected, “I think you come into the CRE in the beginning, and unless you have an established relationship with everybody, you’re a newcomer, you come in and you almost have to prove your worth as a collaborator, and you have to prove that you’re someone who does what they say they’re going to do as well, so you’re a good worker, you’re someone who delivers” (1.2). The inadvertent effect of facilitative interviews around KT and team function was the establishment of communicative trust, based upon the open sharing of information, disclosure and ensured confidentiality.

Similarly, there were times where trust was not activated between investigators. This was often a result of a lack of investment in context (e.g. not participating in opportunities for collaborative decision-making or KT facilitation activities). Investigators were less likely to identify scholars to collaborate with outside of their direct network (i.e. interaction node), regardless of the investigator’s competence. The absence of collaborative arrangements occurring when these networked opportunities were not capitalized upon provided evidence of this relationship. For instance, reflecting on their relationship with other investigators, one CI commented, “I think it’s just that people kind of—we have good intentions about being interdisciplinary, but it’s really, really easy to slip into your comfort zone and just do what you’ve always been doing, and you’re doing your bit of a bigger picture and that’s just fine”.

A key activity of the CRE was the creation of KT resources, which were tangible outputs that built upon earlier work and integrated diverse knowledge across diverse disciplines and stakeholders (e.g. “[developing the KT resource] has sort of served as an integrator, I do not know what the right word is, like a melting pot”, 1.2, graduate student). Invested CRE members, having already established trust and commitment to a shared vision, created a sense of shared ownership over the KT resource and corresponding investment in the process of its development. Here, the KT resource was considered a culmination of investigative efforts (i.e. an output); however, the process of its development enabled investigators to focus clearly on a materialization of this knowledge.

### Valuing collaboration

Governance structures and processes that provide incentives and opportunities for the meaningful participation and connection of team members (including fellows and trainees) lead to an increased willingness and ability to invite contributions from others. This also enables socialization into organizations and established collaborative networks (*mechanism*). As a result, invested team members develop positive perceptions of collaboration and teamwork, including favourable attitudes towards a team-oriented working style, networking, and ability and comfort in bridging diversity (e.g. cultures and diverse stakeholder interests) (*outcome*).

Regularly scheduled CRE meetings provided a platform for collaborative problem-solving and decision-making. However, there were times where team members reflected that these meetings could be more forward-looking and less administrative (e.g. “they’re just reporting meetings it seems to me”, 1.9, CI). Team members valued the opportunity to prioritize initiatives and consider the complexities of next steps for the team but at times felt they lacked the context where these integrative collaborative discussions could take place. In turn, this created a context favourable for the KT facilitation process, which provided opportunities for team connection and incentivized members towards the development of shared KT outputs. As one CI expressed, “[The interview] was good, actually, to think through—because yes, I must admit I haven’t put too much thought, kind of strategic thought into what we do. I just kind of have this general idea that this is what we’re going to be doing, without kind of thinking through” (1.9).

We noted that scheduled events (e.g. management meetings), opportunities for informal social interaction among CRE members, incentives for team members to broaden the focus of their research over the lifespan of the collaboration, and clear points of contact for local and international team members created a favourable context capable of increasing the willingness and capacity to invite contributions from others and enable socialization into organizations and established collaborative networks. As one graduate student commented, “I found the social component, actually face-to-face meeting with people, opportunities for talking, has been really valuable for building connections, understanding what people do, what projects they are working on, what are the features of those projects that are interesting or might be able to tie into work that I am doing or how understanding their work you might be able to then let them into someone else” (1.1). As a result, this fostered positive perceptions of collaboration and teamwork for participating team members. This response included positive attitudes towards a collaborative and team-oriented working style, engagement in networking, the incorporation and consideration of diverse cultures and stakeholder interests, and comfort in dealing with people from diverse backgrounds at all levels in various organizations.

## Negotiating the network

Formal and informal collaborations and distributed leadership increased the ability of team members to negotiate and leverage the network resources, and to build readiness and capacity for collaboration. A critical component of this context was the opportunity to socialize with other team members. In this case, the CRE was distributed across academic institutions in one city, with a number of interstate investigators as well as international collaborators. Investigators in close geographical proximity to one another and to the KT team benefited from informal socialization opportunities, which concurrently activated other mechanisms (such as trust) integral to effective collaboration and knowledge mobilization. Other investigators identified the plausible benefits of having the investigative team in one location: spontaneous collaborations could occur and familiarity between investigators would be improved, thereby strengthening the network and the availability of its resources. As one CI explained, “In the ideal there’d be a CRE office, and we’d be bumping into each other all the time. And then you’d get the kind of instantaneous conversations and things that lead to other things. So, that’s the ideal. But that’s not going to happen. So, what’s the second-best solution to try and—well, it’s very difficult to replicate that” (1.9).

Governance processes and structures permitted different kinds of formal and informal leadership, and opportunities for facilitated reflection and learning. These factors led to team members developing the capabilities, persistence and confidence to leverage their resources and connections to overcome perceived boundaries or structural gaps, facilitate knowledge exchange and achieve greater team coordination, reflected in a greater number of cross-disciplinary collaborations and research outputs over time (Table 3). This was particularly notable for investigators who had the opportunity to develop leadership within their respective programme streams, and for those whose programmes of work had more intuitive similarity. Familiarity with KT and other respective programme arms was challenged by physical distance; those familiar with collaborative research or interprofessional clinical practice more readily achieved synergies between diverse research streams.

**Table 3:** Summary of CRE productivity data (2016–2019)

Productivity measure	Year			
	(1) 2016	(2) 2017	(3) 2018	(4) 2019
Peer-reviewed publications				
First author (%)				
Graduate student	–	5 (33.3)	8 (47.1)	13 (38.2)
ECR	–	4 (26.7)	3 (17.6)	6 (17.6)
AI	–	1 (6.6)	3 (17.6)	4 (11.8)
CI	–	5 (33.3)	3 (17.6)	11 (32.4)
Total citations^a^	–	198	416	337
Collaborative publications^b^ (%)	–	5 (33.3)	8 (44.4)	15 (44.1)
Australian competitive grants (category 1)				
Principal investigator (%)				
Graduate student	–	–	–	–
ECR	–	–	–	–
AI	–	–	–	–
CI	2 (100.0)	–	1 (100.0)	4 (100.0)
Other public sector research income (category 2)				
Principal investigator (%)				
Graduate student	–	–	–	–
ECR	–	–	–	3 (75.0)
AI	–	–	–	–
CI	–	2 (100.0)	2 (100.0)	1 (25.0)
Industry and other research income (category 3)				
Principal investigator (%)				
Graduate student	–	1 (20.0)	–	–
ECR	–	1 (20.0)	–	3 (75.0)
AI	–	3 (60.0)	–	–
CI	2 (100.0)	–	–	1 (25.0)
Awards and recognitions				
Graduate student	–	2 (50.0)	2 (40.0)	6 (46.2)
ECR	–	2 (50.0)	2 (40.0)	4 (30.8)
AI	–	–	1 (20.0)	1 (7.7)
CI	–	–	–	2 (15.4)
Completed postgraduate students	–	–	1	2
Contributions to Commonwealth policy guidelines	–	3	–	3
Contributions to State policy guidelines	–	1	–	4

*ECR* early career researcher (including graduate student and postdoctoral fellows), *CI* chief investigator, *AI* associate investigator

^a^Citations obtained from Google Scholar (9/12/2020)

^b^Collaborative publications were defined as peer-reviewed publications coauthored by CRE investigators from two or more disciplines

### Valuing learning through reflection and trust

Learning about other approaches to research, as well as other theoretical framings and research emphasis, was integral to teamwork within the CRE. While contingent on connection, valuing learning required that the benefits of each approach be demonstrated in relation to the overall research objectives. Here, mentoring opportunities and facilitated reflection, wherein investigators could push beyond their boundaried priorities and disciplinary perspectives, was critical. In this way, facilitated reflection activities, such as arts-based elicitation, with experienced KT researchers *(context)* led to team members being aware of various problem-solving approaches as well as an impact-based and context-dependant framing of the research problem (*mechanism*). This resulted in team members having an outlook that valued reflection, experiential learning, self-appraisal and adaptation, including positive attitudes towards integrating feedback to improve performance, engaging in professional development and self-directed learning (*outcome*).

There was evidence of a lack of reflection on some core tenets of the proposed CRE work at the onset of data collection. KT itself was a concept that had not been considered by all investigators, despite being an underlying framework for CRE. How one’s research programme “fit” into this framework, and how the KT framework would facilitate each stream of research, was often not considered, nor was its relationship to transdisciplinary research (e.g. “I guess there’s…they’re two terms and I assume that they have a slightly different meaning”, CI, 13). This was particularly true for junior investigators and for those researchers without a clinical orientation (e.g. non-clinician-scientists). For example, when asked whether colleagues share similar understandings of KT, one student stated, “I don’t know. I haven’t had a chance to speak to other students how much they know about knowledge translation, so I’m not sure” (1.8). Established clinical researchers were more likely to proclaim a solidified view of KT, hence the need for continued and facilitated contact.

Trust was a key outcome enabled through the continued opportunities for learning, and occurred over time and in different ways depending on the type and relationship of investigators. Established positive relationships among senior team members (i.e. CIs and AIs), time for productive interactions and conversations between team members, and resources and support from leadership for new team members (e.g. graduate students and postdoctoral fellows) to be involved in collaborative projects (*context*) promoted an understanding of how best to work with others in the network to attain mutually agreeable outcomes (*mechanism*). This promoted positive beliefs about the intentions, integrity and character of peers and the team as a whole, including positive attitudes towards the mutuality and reciprocity of working relationships with others, the identity of the group as a whole and the cohesiveness of relationships with peers (*outcome*). Drawing on a mechanical metaphor, a graduate student expressed, “I think trust grows out of doing what you say you’re going to do…that you can rely on someone. If you’re all cogs in wheel or you're part of a bigger system—I want to say gears, but I don’t really know—if it’s an engine, you’ve all got to be doing what you say you’re going to do at the right time; otherwise you get out of sync and the whole thing breaks” (1.2). There was also evidence of trust functioning as a mechanism, wherein trusting relationships developed through transactional exchange, for example, fostered cohesion and more collaborative outputs. However, the presence of trust as an outcome resulting from the awareness of others within a resourced context was particularly well supported by the data.

### Becoming a team leader and boundary spanner

**Fig. 5:**
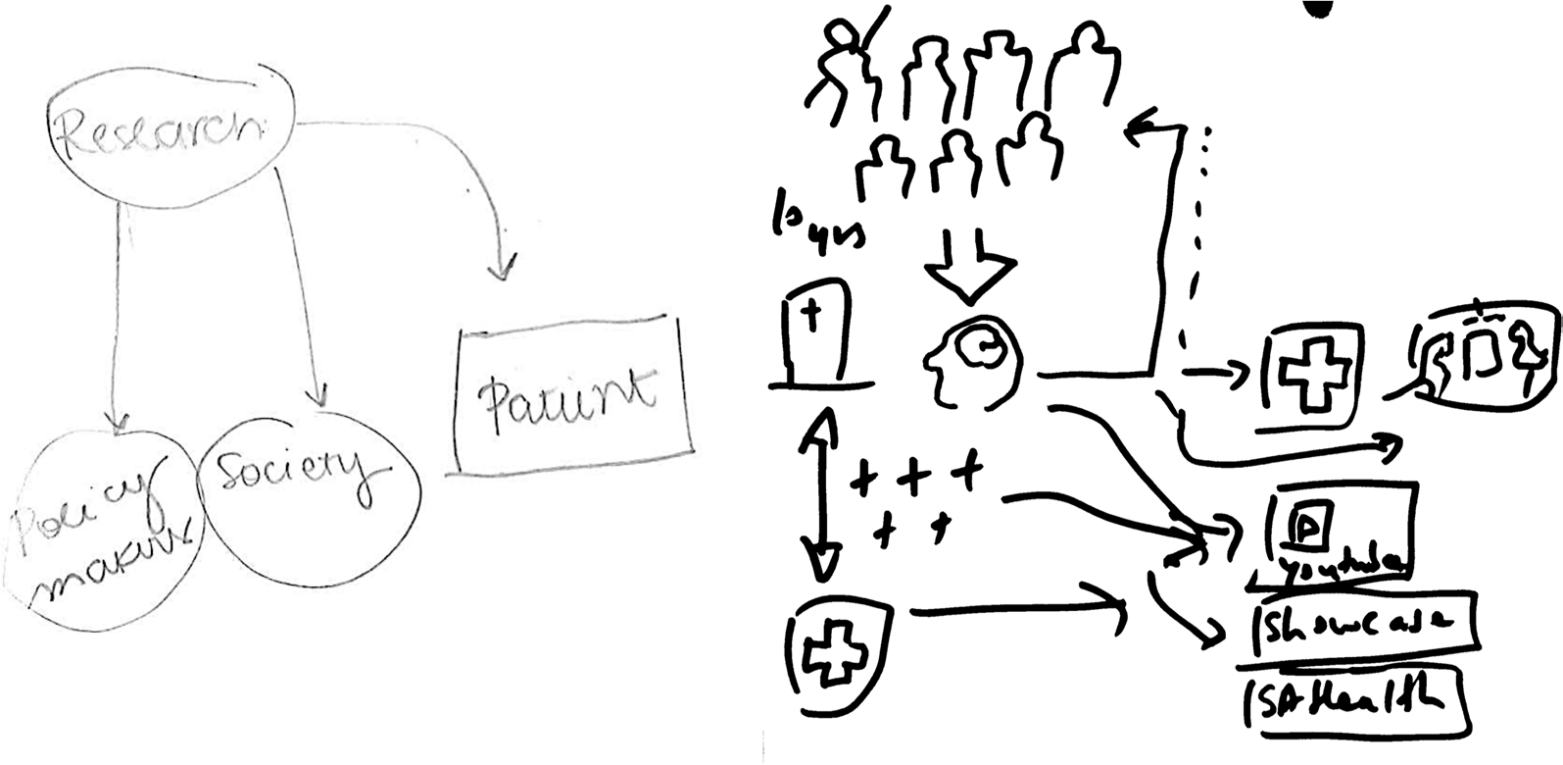
Left: initial depiction of KT process showing linear (unidirectional) movement of knowledge from research to policy, society and patients (graduate student, Participant 1.5, data time point 1). Right: depiction of KT process showing multidirectional relationships and knowledge exchange between healthcare consumers, health services, government and media, with the researcher functioning as the central link or boundary spanner (graduate student, Participant 1.1, data time point 3)

Distributed leadership within project streams allowed for the tailoring of research initiatives aligned with investigators’ strengths, and through trust and autonomy, provided investigators with the flexibility and permission to act as a bridge between stakeholders (e.g. team members, community stakeholders) to facilitate the exchange and negotiation of knowledge. As a CI commented, “Someone has to be proactive, I guess. So, postdoc or CI has to think, ‘Oh, this is something that might be relevant to one of the other streams’, and set up a meeting or initiate contact to stand up discussion about that” (1.9). As a result, CRE members functioned as critical links between evidence and implementation, often reducing the perceived linearity between these concepts. This emerging understanding was reflected in CRE members’ visual depictions of KT and transdisciplinary research processes as iterative and highly interactive (Fig. 5). CRE team members thus developed skills in facilitating knowledge exchange with diverse stakeholders and were better able to identify opportunities to inform policy and research agendas, engage in deliberate dialogue, negotiation and conflict resolution, and work with decision-makers to assess, adapt and apply research findings (outcomes).

Our findings demonstrate that governance structures that enable nonhierarchical leadership also benefit from established KT leadership that exemplifies collaborative, distributed decision-making, as well as opportunities to model such behaviours. The senior KT researcher had developed KT frameworks exemplifying such values; established CRE meetings provided opportunities for this modelling to take place. The nonhierarchical approach was credible due to established expertise wherein multidirectional information-sharing was modelled, encouraged and expected. This in turn increased the connectivity between previously unlinked teams and individuals.

Our findings demonstrate that organizing teams around disciplinary contributions mandated opportunities to monitor the progress of collaborative efforts. Yet, such organization, imbued with some foundational collaborative opportunities and distributed leadership (context), can facilitate a clearer understanding of the team goals (mechanism), leading to individuals being able to use this preliminary understanding as a springboard for further collaborative efforts. In this way, an explicit understanding of the preliminary team goals, with permission and opportunity to work collaboratively to tailor and operationalize these objectives based on context-specific needs and resources, provided opportunities to develop team leadership ability. Leadership ability manifested in the capacity to persuade others and garner buy-in for shared initiatives, the know-how to act on stakeholder views and needs, and the ability to adapt problem-solving approaches and strategies as necessary (*outcome*).

## Mobilizing the network

Mobilizing the transdisciplinary team network required collective efficacy—specifically, the interactivity, coordination and shared beliefs of members that could be leveraged through investigator skills, influence and consistent effort. At the commencement of the CRE, the concentration of KT expertise resided predominantly within the KT project stream “hub”. However, as the broader CRE team members continued to gain exposure, experience and confidence in participating in specific KT activities, and strengthened relationships with team members and stakeholders, they internalized alternative ideas and knowledge about KT relevant to their research. They also developed an awareness of the priorities and needs of different stakeholder groups (e.g. consumers; Fig. 4). This resulted in CRE team members developing the required understandings, skills (e.g. flexibility) and attitudes to lead and involve others in KT activities. This was reflected in an ability of team members to identify KT opportunities, as well as methods to mediate, move and/or adapt relevant knowledge within and beyond the network. Also demonstrated was an accumulation of research impacts—including benefits for one or more areas of society, policy or health—and contributions to the academic knowledge base. Importantly, an increase in collaborative publications and outputs appeared activated by close relationships to the KT team, including participating in facilitation events.

### Understanding KT concepts and research processes

Facilitation events and efforts (e.g. task-oriented meetings) enabled regular contact between investigators and researchers with experience in conducting key KT activities (e.g. implementation, problem identification). Over time, this context—equipped with the resources of formal and informal mentoring from senior team members and researchers knowledgeable in KT theory and implementation—solidified previously “loose” collaborations. Loose collaborations included those collaborations theorized as relevant during initial grant writing or project conceptualization but not yet functionally operational. Facilitation, openness to different ways of thinking and doing, and the associated time invested towards conceptual collaboration helped formalize collaborative relationships and created new opportunities for KT involvement and contribution.

For instance, through facilitated meetings that centred around KT processes and creating bilateral understandings, CRE team members achieved common understanding of KT processes, identified areas for knowledge contributions oriented towards academic and social impacts, and in turn were provided opportunities to participate in collaborative codesign activities with stakeholders or related activities (e.g. codesign of KT resource). As one CI—a clinical researcher—expressed, “At the moment [my understanding] has gone from this amorphous KT way of thinking—this just seems an overkill in a sense of what’s going on—to being comfortable that that cocreation model is pretty much a part of my future” (1.7). A predominant outcome of such facilitation was “buy-in”, wherein investigators closest to the KT team learned about KT, were able to envision its utility to their own research (*mechanism*), and as such were able to influence others in the process (e.g. supervisors). As a result, a facilitative context imbued with the availability of high-level KT expertise enabled investigators who were in close contact with the KT team and associated facilitation events to identify the relevance of KT to the team’s overarching objectives as well as their specific research stream. This generated buy-in as well as an understanding of substantive and process-based KT knowledge. Such understandings included knowledge about developing research questions that are relevant to knowledge users; definitions of KT and related concepts; developing appropriate search strategies; critically appraising the quality of literature from disciplines using various research methodologies; and understanding the relationship between evaluation and research use (*outcome*).

### Becoming a confident transdisciplinary researcher

The resources from the CRE—including the supportive context for CRE team members to participate in constructive interactions with peers and mentors in the network, have their contributions acknowledged and be provided with opportunities for facilitated reflection—prompted individuals to recognize potential and actual synergies between individuals and teams in the network, and individuals’ own contribution to reaching shared goals (*mechanism*). A number of individuals identified that recognizing these potentials increased motivation towards the end individual and collective objectives. Often, the recognition of possible collaborations increased CRE team members’ confidence in their ability to work well with community partners and decision-makers, navigate administrative and political issues relating to decision-making and leadership, and use technologies to facilitate collaboration (*outcome*).

An important component of gaining confidence towards transdisciplinary research centred upon safe opportunities for knowledge exchange between investigators. These opportunities were often predicated on trusting relationships, valuing of individual contribution, and the space and time for such conversations to occur. As one graduate student explained, “I guess, to me, there’s a growing awareness of a potential contribution that my work could make to other projects within the CRE, so that being the main change, perhaps gaining some profile or some greater awareness of my work as I go through, just by producing results and meeting people and collaborating with them, that sort of thing” (1.2). Modelling of collaborative behaviours became important as the CRE progressed; centring collaboration around the creation of a KT resource manifested the unique knowledge contribution of each research stream involved, wherein participation in formalized CRE meetings provided opportunities for a dialogical approach to identifying key outputs and next steps for the research team.

### Learning to disseminate and use knowledge

We identified important contextual factors that helped investigators learn to work adaptively in a manner reflective of diverse standpoints, knowledge bases and shifting priorities and interests of stakeholders (e.g. “you can understand what sort of information would be useful to people in the field”, graduate student, 1.1). These included consistent exposure and invested time to discuss key KT concepts and processes; opportunities to interact with diverse stakeholders to understand diverse perspectives on research concepts and meanings of impact; and governance and institutional support for these interactions to occur. These exposures generated understandings of diverse stakeholder needs and perspectives, informed knowledge of communication preferences of various stakeholder groups and fostered recognition of techniques to communicate concepts for different audiences (*mechanism*). In turn, such exposures helped team members develop the ability to share available knowledge in accessible ways for various knowledge users. Investigators gained skills in summarizing research findings, distributing relevant knowledge to stakeholders and highlighting key messages that could influence practice and decision-making (*outcome*).

The various ways of addressing complex social problems such as the management and impact of frailty on individuals and communities mandated a negotiated and adaptive approach to collective problem-solving. The KT resources of the CRE provided a direction and structure for such conversations to take place. Collaborative arrangements—such as scheduled events with multiple team members, stakeholders and a KT facilitator—allowed the CRE team members to engage in productive dialogue with various stakeholders (e.g. patients, clinicians and decision-makers) across disciplinary and social boundaries. Modelling the process of cocreation with stakeholders and providing timely access to necessary data (*context*) led involved CRE team members to appreciate the needs, perspectives and priorities of stakeholders, improve relationships among stakeholders and facilitate the relevance of existing evidence to address local issues (*mechanism*). This in turn enhanced CRE team members’ capacity to effectively apply existing research findings to clinical and policy situations or to inform further research, including knowing how to interpret data, integrating evidence into practice with specific client populations, sustaining interventions and identifying practical implications for each member’s own research and practice (*outcome*).

## Discussion

In this realist evaluation, we developed and refined our programme theory to explain why, for whom and under what circumstances transdisciplinary research collaboration impacts on KT-related understanding and behaviours in a 5-year NHMRC-funded CRE [1]. Extending earlier investigations of collaboration-based implementation [29] and knowledge mobilization within cross-sector collaborative partnerships [48, 49], we identified three key processes—which we understand as interrelated programme theories—by which transdisciplinary collaboration and KT influence one another. Specifically, our findings demonstrate that under the right contextual conditions, transdisciplinary team members respond through an improved ability to (1) navigate the network, (2) negotiate the network and (3) mobilize the network. These findings build on recent investigations of transdisciplinary research in ageing that highlight the importance of assessing the team’s shared understanding of KT, going beyond research productivity such as counts of shared grants and publications (e.g. Sargent et al. [50]).

Our findings provide insights into the complexities of transdisciplinary team research and underscore the reciprocal relationship between transdisciplinary research and KT. By creating a context favourable to the creation and activation of networks and subnetworks by way of facilitation, trust and leadership, we were able to improve the functioning of the transdisciplinary team while simultaneously cohering and catalysing our KT processes and outputs (Fig. 1). This reciprocity became apparent throughout our analysis and prompted a deeper consideration of the synergistic mechanisms paralleled by both the KT and transdisciplinary bodies of literature. The realist evaluation process served as a guide, allowing us to maintain theoretical awareness throughout the research process and delineate conceptual linkages. Through this process we were able to uncover that the facilitation of a transdisciplinary team using principles from an iKT approach (specifically, the co-KT framework [8]) functioned to improve transdisciplinary team research; indeed, we discovered that these processes are inextricably linked. This was particularly evident for those individuals who operated in close geographical proximity (e.g. were available for frequent meetings and working sessions and hence gained opportunities to navigate and negotiate the network more readily; Fig. 1), who were provided an opportunity for autonomous leadership of a research arm within the overarching transdisciplinary objectives, and through accessible and supportive social structures, capitalized on opportunities to build trusting relationships to further cohere and activate the research network.

The importance of understanding the influence of social structures and relationships within professional networks has been highlighted in the KT and transdisciplinary research literature, respectively [46–48]. In the field of KT and evidence-informed practice more broadly, social network analysis methodologies have been applied to examine patterns of connections or ties among agents within an interconnected group or network [49]. Long and colleagues [49], for example, conducted an online survey to examine the influence of clustering—understood as silos based on geographical proximity, professional proximity/homophily and past experience—on patterns of collaboration in an Australian translational research network. They reported that geographical proximity and past working relationships are key factors in the choice of collaborators in a dispersed network. Future intended collaborations included several weak ties or ties based on reputation, suggesting that the research network provided new opportunities for collaboration. Our findings similarly demonstrate that in a geographically dispersed network such as the CRE, enhancing interactions between team members and facilitating non-project-related social interactions may help build trusting relationships. This finding aligns with knowledge of the critical role of central actors in network dynamics, wherein those individuals who interact with the most other actors aid cohesion and collaboration by improving linkages, often between isolated clusters. Moreover, key network players might have an important brokerage and boundary-spanning role in crossing physical, organizational and cultural (e.g. discipline-specific) boundaries to exchange knowledge and facilitate linkages between groups [24]. In our future work, we will use social network analysis to evaluate how the social structures and relationships in the CRE influence KT outcomes such as knowledge-sharing, network size and research collaboration.

Our findings present a picture of a committed transdisciplinary team that, while imbued with expertise in KT and frailty research, was inexperienced in the complex processes of transdisciplinary team research. As KT researchers leading this programme, we considered in our preliminary member consultations and data collection cycles members’ baseline understanding of both KT and transdisciplinary research. The corresponding visual and narrative data generally emphasized the importance of network connections, as well as the bringing together of multiple viewpoints, to transdisciplinary team research (e.g. visual image of round table with multiple chairs around it; image of multiple lines connecting dots within a circle). These components were considered integral to creating more ideas (or solutions) with possible social impacts. While not inaccurate, this exercise demonstrated an understanding of transdisciplinary research that was preliminary at best; the complex and established processes of transdisciplinary research practice were not recognized [2]. Given that such processes are integral to the integrity, function and impact of a transdisciplinary research team, their omission created an environment open to the facilitative aspects of KT leadership within the team. The uncertainty of this unknown relationship mirrored the many uncertainties experienced by CRE members as they progressed, on varying paths, towards different understandings of KT and its processes [51]. Since “uncertainty is socially constructed” [52] (p. 15), there exist opportunities to reconstruct new understandings through the mechanisms of transdisciplinary team work.

For instance, a critical foundational component of transdisciplinary research involves conceptual ground-working, where a shared conceptual model is established as a means of integrating diverse sources of disciplinary knowledge [2]. Within the CRE, this work was not specifically done under the guise of transdisciplinary work but rather, was conducted under the KT research umbrella. This contingency may be unique to KT predicated on coproduction of knowledge and the transdisciplinary research relationship, given their parallel orientation to solving societal problems [53]. Through one-to-one meetings, facilitated group sessions and CRE trainee talks, the terminology, processes and objectives of KT were engaged with—often repeatedly and conversationally—until a shared understanding was attained. These activities, captured in Fig. 1 within the mobilizing the network domain and in the key milestones of the CRE lifespan table (Table 1), helped create a sense of shared vision within the CRE, which is an essential aspect of successful transdisciplinary team function [2, 52–54]. This process reflects a view of integration as a thought collective between group members that guided programme development and subsequently fed back insights to further refine the thought collective [53].

A further consideration encountered was that the inclusion of a KT framework, specifically one that values collaboration and the democratization of knowledge, greatly influenced how investigators in the CRE considered and appreciated different sources of knowledge. Ultimately, this manifested as a shift in what constituted “legitimate” knowledge. The democratizing of knowledge is what collaborative and iKT approaches are capable of achieving. As our CRE was predicated on a broad yet well-accepted definition of knowledge, this allowed a starting point for us to further democratize knowledge and appreciate the influence of networks in this process.

Similarly, transdisciplinary research often involves tailored activities aimed at improving the disciplinary integration of knowledge. The creation of boundary objects is one such integrative activity. As Bergmann et al. [2] indicate, “Materialization is the basis of the integration effect of artifacts. One could call them integration interfaces that have become material. Their concreteness and vividness promote cognitive integration... they build bridges not only across scientific fields and disciplines but also from within the sciences to non-scientific stakeholders” (p. 106). Within the CRE, the collaborative creation of KT resources (e.g. Frailty: Every Step You Take Matters) [37] effectively functioned as boundary objects—integrative artefacts, the “focal point of joint efforts” [2] (p. 126). The focal activity of KT resource codevelopment enabled the integration of diverse disciplinary knowledge as well as the crystallization of such knowledge across disciplinary *and* methodological traditions [37], enabling investigators to consider the interrelationship of knowledge contributions while providing a sense of achievement in the consideration of social impacts. This is akin to what Kania and Kramer [54] refer to as collective impact: the commitment of diverse actors towards a particular social agenda achieved through centralized infrastructure, dedicated staff and structured processes leading to mutually reinforcing activities for participants, among other outcomes.

The codevelopment of KT resources as an integrative activity facilitating KT understandings and behaviours as well as transdisciplinary research processes also served as a method for integrating scientific and societal bodies of knowledge—a critical component of both transdisciplinary research and those iKT approaches seeking to meaningfully involve stakeholders throughout the process [2, 7]. Indeed, the co-KT framework guiding our transdisciplinary efforts emphasizes the democratization of knowledge through interactions between social and scientific actors through an integrative “middle ground” [8]. An authentic consideration of societal perspectives and priorities served as a motivator for disciplinary knowledge integration [2], suggesting a shared mechanism between collaborative KT and transdisciplinary research motivations, which can be activated through a favourable research context (e.g. trust, democratic leadership).

Our findings make an important contribution to understanding the impact of distributed and flexible leadership on transdisciplinary team function, with attention to career stage and role autonomy. Research leads (e.g. CIs) that adopted a distributed leadership style empowered junior investigators (AIs and fellows) to shape their respective activities in a manner that played to their strengths; such flexibility is integral to the unforeseen opportunities made possible through transdisciplinary collaboration. Such opportunities and resulting directions are largely emergent; hence, distributed and flexible leadership appears to be a critical mechanism for network negotiation within a transdisciplinary team. The democracy and fluidity of networks appears contingent upon this leadership style and is an important asset to both transdisciplinary research and KT processes. As such, our findings suggest that imbuing a transdisciplinary team with KT leadership—specifically an open, flexible and distributed KT leadership style situated within an integrated or co-KT approach—has implications for transdisciplinary leadership, and exerts benefits across both processes (i.e. KT and transdisciplinary research).

When central actors adopt a distributed leadership style as was documented in the current study, tasks associated with cognitive (e.g. visioning, framing), structural (e.g. coordination and information exchange) and process-based (e.g. activities to promote constructive and productive team interactions) dimensions of leadership may be enabled [25]. This style appears particularly critical when the transdisciplinary team itself is geographically dispersed, requiring a higher extent of brokerage and boundary-spanning for productive collaboration to occur [25]. Indeed, geographical proximity impacts collaborative ties extensively, with actors at one location generally experiencing more collaboration through improved mobilizing, networking and negotiating opportunities within the network (Fig. 1) [49]. Our work demonstrates that while geography was important to collaboration, this “practical incentive” [24] (p. 7) can be mitigated through facilitation aimed at improving the complex exchange of knowledge. This can be facilitated through the creation of multiple leaders (formal and informal) within programme arms. While this work responds to current limitations in understanding leadership behaviour in complex networks identified by others [24], future research examining the tenets of transdisciplinary leadership is warranted.

Our results suggest that transdisciplinary research and KT skills and techniques work in concert to promote success in both fields. While the broad goal of transdisciplinary research is to optimize the generation of new knowledge by bringing teams together to think and problem-solve in new ways, KT is about optimizing the spread and impact of new knowledge by bringing stakeholders together to think and find implementation solutions [26]. Our research demonstrates that the intellectual and interpersonal skills and techniques required for both activities draw upon similar sources (the ability to leverage social network features and processes). The consequence of this is that the stronger a network member becomes in transdisciplinary research, the more likely they are to be successful in KT if both activities are made more explicit. Individuals engaged in transdisciplinary research, when also exposed to explicit KT activity, are better able to navigate, negotiate and mobilize the networks in which they operate.

A limitation of the current study is that we studied one CRE over its lifespan and therefore do not make claims about how our findings are representative of other transdisciplinary teams. We have endeavoured to provide a detailed description of the composition and activities of the CRE so that readers can determine the theoretical transferability of the results to collaborative teams working in other contexts. Additionally, our literature review was limited to articles published in English, and a majority discussed discrete aspects of connecting knowledge production to knowledge use and/or team-based research; they did not explicitly focus on the intersection between transdisciplinary and KT. While useful to indicate how the CRE structure changed over time, our figures of the CRE internal structure (Figs. 2, 3, 4) are presented as general visual representations pointing to the CRE internal structure rather than reliable data points informed by social network analysis, as will be completed in our future work. Lastly, complexity theory encourages us to generate sets of principles or rules that can be applied by individuals within dynamic contexts. Arguably, the three network mechanisms or principles we have generated are necessarily dynamic (i.e. drawing on all CMO configurations) because they reflect the complexity of transdisciplinary and KT activity within complex adaptive systems. The methodological question, then, is whether realist evaluation can be used to generate principles that can help us better handle the uncertainties of working with complexity. Future research could combine realist evaluation with other tools and methods such as social network analysis, stakeholder analysis, participatory research and systems mapping to examine activities and relationships in complex adaptive systems [17].

## Conclusion

The relationship between transdisciplinary research and KT is contingent, reciprocal and strongly influenced by contextual factors such as governance structures, leadership styles, time and the availability of expert facilitation. In the absence of formal training in transdisciplinary research approaches, embedding and facilitating KT—specifically integrated, collaborative and complexity-based KT approaches—can promote the function and collaborative success of a transdisciplinary research team. Transdisciplinary research and KT are, at a minimum, connected by an *impact mandate*: solutions to real-life problems are the objective, achieved through the deliberate bringing together of investigators or activities to move knowledge into practice or policy. Our findings however emphasize that both transdisciplinary research and KT are experienced as complex networks: contextual resources must be available to help investigators navigate, negotiate and mobilize these evolving networks. Working within these networks activates an appreciation of diverse knowledge, enables the democratization and accompanying legitimization of knowledge, and poses integral challenges and potentials for transdisciplinary leaders tasked with facilitating this predominant objective. Whether this in turn generates the types of knowledge necessary to tackle complex social problems remains of salient interest.

## Supplementary Information

**Additional file 1.** Overview of facilitation activities.

**Additional file 2.** Search strategy for PubMed (Medline) database.

**Additional file 3.** PRISMA flow chart.

**Additional file 4.** RAMESES II reporting standards for realist evaluation.

## Data Availability

The datasets generated and analysed during the current study are not publicly available since participants did not consent to have their data be in the public domain. Additional information about the data and materials can be made to the corresponding author.

## Abbreviations

AI: Associate investigator
CI: Chief investigator
CIHR: Canadian Institutes of Health Research
CMO: Context-mechanism-outcome
co-KT: Cocreating knowledge translation
CRE: Centre of Research Excellence
iKT: Integrated knowledge translation
IPT: Initial programme theory
KT: Knowledge translation
KT-cnm: Knowledge Translation Complexity Network Model
NHMRC: National Health and Medical Research Council
PRISMA: Preferred Reporting Items for Systematic Reviews and Meta-Analyses
