# Supplementary material for: How transdisciplinary research teams learn to do knowledge translation (KT), and how KT in turn impacts transdisciplinary research: a realist evaluation and longitudinal case study
Source: Health Res Policy Syst. 2023 Mar 21;21:20. doi: 10.1186/s12961-023-00967-x (PMC10032009; doi:10.1186/s12961-023-00967-x)
Supplement: Supplementary file 1 — Additional file 1. Overview of facilitation activities. [file 12961_2023_967_MOESM1_ESM.docx]

**Additional File 1. Overview of facilitation activities.**

| **Activity** | **Date** | **Notes** |
| --- | --- | --- |
| CRE management meeting, mentoring committee established | February 2017 | MA involved in mentoring subcommittee, notes previous experience as mentor/mentee (Minutes, Feb 2017 CRE Management Meeting) |
| Data collection time point 1 | February – April 2017 | Interviews and visual elicitation conducted by MA with n=16 CRE investigators either face-to-face or via Zoom |
| CRE planning workshop | April 2017 | AK and MA provide an update on the main directions for KT: Internal focus is on a realist theory driven evaluation of transdisciplinary research and KT within the team with the idea that “good transdisciplinary research makes for better impact”. AK and MA working on several projects in a collaborative and supportive capacity, e.g., GP/PN study. The following strengths, opportunities and challenges were highlighted. Team strengths: positive team engagement and positive collaborative energy and willingness and a sense of how KT can be involved Opportunities: more face-to-face work, more project opportunities around awareness and strategic consumer engagement. Challenges: Capacity of staff. The path of least resistance aspect, meaning natural alliances due to how the work fits may not produce the most groundbreaking work and we should sometimes work in the more awkward matches of groups to see what develops. This field (frailty) has significant ambiguity and complexity of the concepts should be respected and not be underestimated.  AK produced a visualisation of multidimensional factors of frailty and circulated copies to the group. It showed how each factor of frailty related to each other and enabled a map to overcome the rigidity of using any one particular tool for frailty screening. Because it is a complex flexible and movable construct a single tool may be too harsh and crude to represent this so perhaps a diagnostic tool that was more dynamic could help investigators work out what package of assistance is required. AK expanded: The collection of the story is how we may gather the data and these multidimensional points are the beginning coordinates on a map that you can populate from both a narrative, plus clinical examination, plus other sources. Imagine a patient who told the clinician their story and the clinician then pulled the relevant data from that story, so the patient would leave that encounter with a summary sheet with a diagram of themselves against the multidimensions.  (Minutes, April 2017 CRE Planning Meeting) |
| ML joins KT team, MA commences maternity leave | June 2017 | MA commences maternity leave. New KT team member (ML) formally introduced to group at June 2017 CRE management meeting, advises he will be picking up MA’s work around KT and supporting other CRE projects. ML speaks about his background in psychology and qualitative research methods. ML advises that the KT team has completed the first round of data collection and are currently working through CRE staff/student interviews. |
| CRE management meeting | October 2017 | AK and ML provide update on KT theme – preparing to present RE protocol at Realist Research Evaluation and Synthesis Conference (October 2017, Brisbane, QLD) and Knowledge Utilisation Conference (November 2017, Melbourne, VIC)  (CRE management meeting minutes, October 2017) |
| CRE management meeting | March 2018 | AK queries: “we need to re-examine what we want to achieve by 2020 and how we have aligned or moved away from that. Also whether we should consider other sources of income and how we tap into those”.  AK: “Language is very important in the delivery of assessments and education. We need to use our student teams to change the language used”.  AK- we have the centre for digital health technology- Prof Anthony Maeder. At the next meeting perhaps we can host it at Flinders and have a show and tell as there is some synergy and opportunity for different interdisciplinary teams to get together.  ACTION: July 2018 CRE Management meeting relocated to Flinders University (site of KT team). |
| Data collection time point 2 | March – June 2018 | Interviews conducted by MA and/or ML with n=9 CRE investigators either face-to-face or via Zoom |
| CRE KT planning workshop | July 2018 | MA and ML facilitated planning workshop at Flinders Victoria Square. Agenda as follows:  Purpose and background for the session  Many of you have participated in our internal study of transdisciplinary research and knowledge translation. Through this study we have identified:   - CRE investigators greatly value coming together to share ideas, but find it can be difficult to have the space and dedicated time to do this - Investigators share a common desire for opportunities for brainstorming and knowledge sharing. - KT matters to everyone and relates to all ongoing CRE research. A number (the majority) of investigators have identified a KT outcome as integral to the CRE shared vision.   As such, we wanted to take this opportunity to bring strengthen our collaborative efforts towards further transdisciplinary collaboration, while simultaneously strengthening our KT efforts at this important point in the CRE’s lifespan.  A collaborative approach to planning and KT fits with the KT framework that we adopted from the outset of the CRE work.  The purpose of the workshop is to:   1. Contribute your ideas about key priorities/goals (e.g., raising public awareness about frailty) and brainstorm ways in which different research streams tie into these identified aspects; and (ii) begin to identify research evidence from across the CRE that can be integrated and mobilised to form the context of a collaborative, evidence-based implementation strategy. 2. This is particularly timely considering the recent Rapid Applied Research Translation for Health Impact Grant Scheme via SA academic health science and translation centre (Medical Research Future Fund MRFF call) circulated today by CRE Principal Investigator.   **Plan for today:**   - Small group activities and facilitated discussion as an interactive planning workshop |
| Consumer focus group workshop | September 2018 | MA, ML, and AK present findings of consumer focus group research to CRE management group. Part 1: consumer findings, outline of key themes, key messages, and actionable items. Part 2: Next steps, discuss resource development (approach, avenue, integration of CRE evidence), identify affiliated work plan and next steps (informing interventions and projects, HCP education, continued arts-based work, e.g., PRAXIS artspace exhibition of arts-based KT work) |
| CRE management meeting | September 2018 | Update from KT workshop: MA outlined the desire to undertake video development based on the comments by Prof John Morley during the review process. RV advised there was no specific budget for a video production within the original plan but advised that in the CRE budget there is a small amount of budget remaining under Screening Pathways – Project 2, Screening Pathways – Project 3 and Screening Pathways Knowledge Translation.   - - Suggested using a small portion of the remaining funds to leverage further funds from a partner such as the Office of the Ageing (who have previously done an exercise video).   - Suggested the CRE apply for THRF Translation grant as this is would be a rapid translation type of project   MA stated that funds would be used to make a quality pilot material and leveraged to make something systematic and broader reaching. MA is considering a structured roll out with a short turn around to a deliverable in order to then develop something larger scale using the framework available through Flinders. MA identified the video using consumer narrative as a potential integration point for the other evidence developed from the CRE. AK clarified that MA would use this work as an opportunity to pilot these ways of communicating these messages and she would be working collaboratively and taking steps to ensure the quality, interactivity and impact of the medium. RV strongly suggested MA meet with Office of the Ageing (OFTA) and also with THRF to outline the importance of the work, advise that we are coming to the table with $30k and request that they contribute.  AK was supportive of using the $30K Screening Pathway Knowledge Translation funds and felt it was important to structure the communications in meetings with Office of the Ageing and THRF to show it is a significant piece of work. AK continued commitment to work with MA and ML to develop ideas and to meet back with the CRE team to ensure the planned work is appropriate.  RV suggested AK contact OFTA with the chief nursing officer because they are highly active in SA about quality of care and the new working standard “Comprehensive Care Standard”. AK has already had a chat with PF (THRF) who is visiting Flinders so she will approach him in more details then. PF asked who the audience would be- Would older people be looking at the videos?   - RV advised consumer videos can be used for education of students, presentations, educating the scientific community etc. - MA advised videos with narrative stories engage well with older people - MA expects having narrative script interspersed with the evidence and involving consumer input. - MA planned for multiple structured planned videos. |
| Video-based resource co-design workshops | December 2018-March 2019 | MA involved CRE investigators and consumer representatives in 4 workshop-style meetings to co-design animation and narrative videos. Refer to co-design manuscript for approach (Archibald et al., 2021.). |
| PRAXIS exhibition | ~April 2019 | MA and team exhibit artistic work informed by CRE research at PRAXIS gallery, Bowden; CRE team invited to opening. Refer to accompanying manuscripts for details of development (Archibald & Blines, 2021) |
| CRE management meeting | May 2019 | MA: Animation link complete, narrative video almost finished.  RV suggested next Management meeting Strategic Planning for future of CRE- All agreed to assist in understanding what we have learned AK will add a question pertinent to transdisciplinary work.   - - - What do we think we have learned?     - Where are the gaps?     - Each group List of papers (complete or under development) 1 month prior to the next CRE management meeting   MA suggested writing a group reflection piece to highlight transdisciplinary learning and collaboration.  ML and MA included a follow up question on lessons learned.  MA and her team have undertaken community engagement and resource work.  Flinders University has acquired some of the pieces of art from Beyond Measure. Hosted 2 community engagement events and took the winning performance work, “Untangling Frailty” on tour in Yankalilla.   - Academic presentation, Dance work, workshop in the afternoon which was very well received. - Jenny Elliot - CEO of Arts Care (arts health organisation) and her daughter Francesca (Media Studies) produced a documentary of the remote event. - Significant interest and enthusiasm for the work.   Arts and Health Round table 45 delegates from across SA- Government, practitioners, people on ground, Magill house delegates came through.   - Round table had a lot of theoretical discussion and a good response.   MA presented   - John Beard - A&ECS meeting. |
| Data collection time point 3 | February – March 2020 | Interviews conducted by ML and MAPP with n=8 CRE investigators either face-to-face or via Zoom |
| Presentation of interim findings of realist evaluation at CRE management meeting and advisory group meeting | September 2020 | AK, ML, and MAPP presented the interim findings of RE to CRE investigators and subsequently to the CRE advisory group (Presentation title: “Insight into the CRE Transdisciplinary Process: How We Have Evolved”) |
| Final CRE research report (2016-2019) | September 2020 | Final CRE Research Report and infographic developed with assistance from KT team (gathering CRE investigator CVs, requesting information about indicators of research impact, e.g., #contributions to policy guidelines, #research projects involving consumers) |

CRE= Centre of Research Excellence; KT= Knowledge Translation; GP/PN = general practice/practice nurse; RE= Realist Evaluation; QLD= Queensland; VIC= Victoria; SA = South Australia; MRFF= Medical Research Futures Fund; THRF= The Hospital Research Foundation; A&ECS= Age and Extended Care Research Meeting.
