# Supplementary material for: How transdisciplinary research teams learn to do knowledge translation (KT), and how KT in turn impacts transdisciplinary research: a realist evaluation and longitudinal case study
Source: Health Res Policy Syst. 2023 Mar 21;21:20. doi: 10.1186/s12961-023-00967-x (PMC10032009; doi:10.1186/s12961-023-00967-x)
Supplement: Supplementary file 2 — Additional file 2. Search strategy for PubMed (Medline) database. [file 12961_2023_967_MOESM2_ESM.docx]

**Additional File 2. Search strategy for PubMed (Medline) database.**

| **Search platform: PubMed** | | |
| --- | --- | --- |
| **Search terms:** | | |
| **Knowledge Translation** | **Transdisciplinary Research** | **Other** |

| “translational medical research”[mh]  OR  Knowledge translation[tiab]  OR  engaged scholarship[tiab]  OR  implementation scien*[tiab]  OR  knowledge exchange[tiab]  OR  knowledge mobili*[tiab]  OR  knowledge transfer*[tiab]  OR  research translation[tiab]  OR  translational health*[tiab]  OR  translational medic*[tiab]  OR  translational research*[tiab]  OR  translational scien*[tiab] | “Interdisciplinary studies” [mh]  OR  High performing teams[tiab]  OR  Interdisciplinary collab*[tiab] OR  Intersectoral collaboration[tiab]  OR  Intersectoral cooperation[tiab]  OR  Transdisciplin*[tiab]  OR  Team scien*[tiab]  OR  Team research*[tiab]  OR  “Interdisciplinary communication”[mh] | English[la]  AND free full text[sb] |
| --- | --- | --- |

Pubmed was searched using the above terms specific to knowledge translation and transdisciplinary research.
