# Supplementary material for: How transdisciplinary research teams learn to do knowledge translation (KT), and how KT in turn impacts transdisciplinary research: a realist evaluation and longitudinal case study
Source: Health Res Policy Syst. 2023 Mar 21;21:20. doi: 10.1186/s12961-023-00967-x (PMC10032009; doi:10.1186/s12961-023-00967-x)
Supplement: Supplementary file 3 — Additional file 3. PRISMA flow chart. [file 12961_2023_967_MOESM3_ESM.docx]

**Additional File 3. PRISMA flow chart.**

Records removed before screening:

Duplicate records removed

(*n* = 409)

Records identified from PubMed, CINAHL, and Scopus

(*n* = 1584)

July 2000-December 2019.

**Identification**

Records screened

(*n* = 1175)

Records excluded

(*n* = 1092)

**Screening**

Reports excluded: (*n* = 31)

Article is not relevant to knowledge translation and/or collaborative research (*n* = 18)

Article does not report on participants collaborating with a team from ≥1 discipline (*n =* 10)

Article not in English and/or full text unavailable (*n* = 3)

Reports assessed for eligibility

(*n* = 83)

Reports identified from reference lists and grey literature

(*n* = 40)

**Included**

Studies included in review

(n = 92)

Records identified through search of PubMed, CINAHL (Cumulated Index to Nursing and Allied Health Literature), SCOPUS, reference mining and grey literature searching.
